# Asthma: a complex disease from causes to phenotype

**DOI:** 10.1016/j.ebiom.2024.105022

**Published:** 2024-02-14

**Authors:** eBioMedicine

Asthma was first officially named as a specific respiratory disease by Hippocrates circa 450 BCE, and its recognition can go as far back as ancient Egypt. Although humans have known about this disease for a long time, it still has a substantial global burden without sufficient control and management.

According to the GBD 2019 Diseases and Injuries Collaborators, asthma affected an estimated 262 million people in 2019 and increased by 15.7% from 2010 to 2019. In a Global Asthma Network Phase I cross-sectional study published in *The Lancet Global Health* in February, 2023, 6.3% of children, 7.9% of adolescents, and 3.4% of adults who were included were living with clinician-confirmed asthma. In all age groups, asthma control was worse in low-income countries (LICs) and lower-middle-income countries (LMICs) than in high-income countries (HICs). For example, the proportion of children with poorly controlled asthma was 19.9% in HICs, but in LMICs and LICs it was 40.1%. For asthma management, the proportion of individuals with severe asthma symptoms who did not use a corticosteroid-containing inhaler in the previous year was high in all age groups (44.8% of children, 60.1% of adolescents, and 55.5% of adults), and this proportion was even higher in children and adults in LMICs and LICs (65.4% of children and 65.1% of adults). Thus, improved asthma control, with more affordable inhaled medicines, is urgently needed, especially in low-income regions.

The causes of asthma vary and interact with each other, among which environmental factors have a substantial effect. In the April, 2023, issue of *Environ Health Perspect*, J Bi and colleagues reported the association between acute exposure to ambient air pollution and asthma emergency-department visits in ten US states during 2005–2014. They found that increases in cumulative exposure to air pollutants, including particulate matter (PM), major PM_2.5_ components, and major gaseous pollutants, were positively related with increased rates of asthma emergency-department visits; most pollutants had stronger effects on children (ie, younger than 18 years) than on adults. Furthermore, long-term exposure to air pollution was reported to increase asthma symptoms. In a study published in the January, 2023, issue of *Thorax*, M Keirsbulck and colleagues explored the association between air pollution and an asthma symptom score in the French population-based CONSTANCES cohort. The score (ranging 0–5) was based on the number of self-reported symptoms of asthma in the past 12 months (ie, breathless while wheezing, woken up with chest tightness, attack of shortness of breath at rest, attack of shortness of breath after exercise, and woken up by attack of shortness of breath). They observed that the ratio of mean score was 1.12 (95% CI 1.10–1.14), 1.14 (1.12–1.16), and 1.12 (1.10–1.14) per one interquartile-range increase of annual exposure of PM_2.5_ (4.86 μg/m^3^), black carbon (0.88 10^−5^ m^−1^), and nitrogen dioxide (17.3 μg/m^3^). Each of these three pollutants were also significantly associated with each one of the five asthma symptoms.

As asthma is characterised by inflammation and narrowing of the small airways in the lungs, that air pollutants can trigger its symptoms is not surprising. However, exposure to other types of environmental pollutants, such as perfluoroalkyl substances, can also be associated with asthma, even across generations. A Danish COPSAC2010 cohort study published in *eBioMedicine* in August, 2023, showed that increasing maternal exposure to perfluorooctanoic acid (PFOA) and perfluorooctane sulfonic acid (PFOS) was associated with increased risk of a specific low prevalent, non-atopic asthma phenotype by age 6 years in offspring, but not of atopic asthma, asthma exacerbations, or lung function. They also observed an isolated, statistically significant, cross-sectional association between maternal PFOA and high-sensitivity C-Reactive protein (hs-CRP; log transformed) at gestational week 24 (β coefficient 0.15; p = 0.028) and a borderline association between PFOS and hs-CRP, suggesting that hs-CRP might have a mechanistic influence on the development of non-atopic asthma. However, the interpretation of this result should be cautious due to the low numbers of both people with non-atopic asthma and people with atopic asthma. In addition to pollutants, climate change also contributes to asthma. In the June, 2023, issue of *European Respiratory Review*, F Makrufardi and colleagues conducted a meta-analysis to explore the association between extreme weather and asthma. They observed that an increase in extreme weather events increased risks of general asthma outcomes, including asthma events (risk ratio [RR] 1.18, 95% CI 1.13–1.24), asthma symptoms (1.10, 1.03–1.18), and asthma diagnoses (1.09, 1.00–1.19); children and female adults were more susceptible to extreme-weather-associated asthma events. Climate change can also affect airborne allergens, which affect asthma indirectly. A Review published in *eBioMedicine* in July, 2023, summarised how climate change influences airborne allergen seasonality, production and atmospheric concentration, allergenicity, and geographical distribution. The authors also proposed three mitigation approaches: automated real-time airborne allergen monitoring, airborne allergen forecasting and modelling, and smartphone-app-supported health care. These studies provide further evidence as to the ways climate change can affect human health and encourage people to find solutions to control asthma.

The complexity of asthma is not only due to its diverse causes, but also its heterogeneous manifestations. For precise treatment, identifying biomarkers to predict its severity and subtype is crucial. In the January, 2023, issue of *Allergy*, S Vázquez-Mera and colleagues analysed the different expression of serum exosome microRNAs from 30 healthy donors and 119 patients with different asthma endotypes (ie, T2^high^-atopic, T2^high^-non-atopic, and T2^low^) and severity degrees (ie, mild and moderate–severe). They proposed a logistic-regression model with variables of BMI, ratio of forced expiratory volume in 1 s to forced vital capacity (FEV_1_/FVC), blood lymphocytes, monocytes, eosinophils count, and miR-126-3p to predict the severity, which had area under the curve (AUC) 0.90 (0.83–0.96), with 0.84 in specificity and 0.88 in sensitivity. To identify a biomarker for eosinophilic or neutrophilic asthma, K Asamoah and colleagues applied high-resolution proteomic profiles in serum and sputum samples collected from 686 participants with mild–moderate asthma or severe asthma from the U-BIOPRED and ADEPT cohorts. In this study, published in the January, 2024, issue of *eBioMedicine*, the authors found 13 serum proteins associated with eosinophilic asthma; inclusion of all 13 proteins in the logistic-regression model yielded AUC 0.83 (0.82–0.83) to predict eosinophilic asthma. They also identified 12 serum proteins associated with neutrophilic asthma; inclusion of these 12 proteins in the logistic-regression model yielded AUC 0.91 (0.90–0.92). This study provided a non-invasive method for asthma subtyping and was also suggestive to understand distinct mechanisms underlying different asthma phenotypes.

Asthma is a major non-communicable disease. Our understanding of its aetiology, phenotype, and mechanism is still not sufficient. Improved management of people with asthma, accurate diagnostic and stratification biomarkers, and effective therapies are urgently needed. *eBioMedicine*, as a translational publishing platform, welcomes all basic and clinical research on asthma.

